# In vitro study on chondrogenic differentiation of human adipose-derived stem cells on treated bovine pericardium

**DOI:** 10.3906/biy-1908-10

**Published:** 2019-12-13

**Authors:** My Thi Ngoc NGUYEN, Vu Nguyen DOAN, Ha Le Bao TRAN

**Affiliations:** 1 Department of Physiology and Animal Biotechnology, Faculty of Biology and Biotechnology, University of Science, Vietnam National University, Ho Chi Minh City Vietnam; 2 Laboratory of Tissue Engineering and Biomedical Materials, University of Science, Vietnam National University, Ho Chi Minh City Vietnam

**Keywords:** Bovine pericardium, scaffold, adipose-derived stem cells, chondrogenic differentiation, cartilage regeneration, augmentation rhinoplasty

## Abstract

Bovine pericardium has been proposed as an available material for tissue engineering and bioprosthetic reconstruction. In this study, bovine pericardium was fabricated into a scaffold for culturing and chondrogenic differentiation of human adipose-derived stem cells (hADSCs). Bovine pericardium was treated in 10 mM Tris-HCl and 0.15% SDS, followed by crosslinking in 0.1% glutaraldehyde. Treated bovine pericardium (tBP) was characterized as a slight yellowish thin membrane with enhanced tensile strength and strain property. The membrane maintained stability under enzymatic conditions for up to 16 days of incubation. The results confirmed tBP as a cell-friendly scaffold for hADSCs due to low cytotoxicity and its ability to support an appropriate attachment and proliferation of hADSCs. Moreover, there was an accumulation of the extracellular matrix proteoglycan in tBP seeded with hADSCs after 7 and 14 days of chondrogenic induction. *COMP* as a specific marker of chondrogenesis was detected after 7 days, whereas type X-a1 collagen (*Col10a1*) expression was stable up to day 14. However, minor expression of aggrecan was found. Taken together, these results indicate that tBP is a potential scaffold for hADSCs for cartilage tissue engineering.Key words: Bovine pericardium, scaffold, adipose-derived stem cells, chondrogenic differentiation, cartilage regeneration, augmentation rhinoplasty

## 1. Introduction

Augmentation rhinoplasty is known as a procedure in plastic surgery in which autogenous tissues or implants are inserted into the patient’s nose structure to correct physical problems (e.g., congenital disabilities, trauma, infection, excessive reduction rhinoplasty, or submucosal resection) (Nguyen et al., 2015) or purely for aesthetic reasons. Autografts harvested from conchal cartilage, septal cartilage, and rib cartilage are commonly used in augmentation rhinoplasty (Herman and Strauch, 2008; Nguyen et al., 2015; Wee et al., 2015). However, it is insufficient in amount and associated with morbidity and scarring at the donor site and additional operation time. Other options for these limitations of autogenous cartilage tissue would be the use of available commercial allografts (e.g., human cadaver rib cartilage – Tutoplast), xenografts (e.g., porcine skin, bovine pericardium – Tutopatch), and alloplasts (e.g. silastic (silicone), Gortex (ePTFE), injectable fillers (hyaluronic acid, CaHA, Aquamid)) (Malone and Pearlman, 2015). Whichever grafts are used, there are still reported issues related to the potential warping and visibility of grafts through the skin, which may cause a deviated and hard appearance in the nasal contour. In addition to the current grafts, commercial bovine pericardium (Tutopatch) has been introduced to disguise the graft and allows it to be molded to generate a smoother and more natural shape for the nose (Nguyen et al., 2015).

Cell-based tissue engineering provides a novel alternative solution in which autologous chondrocyte implantation has been applied for repairing damaged cartilage (Madeira et al., 2015). Chondrocytes can be harvested and cultured to obtain an appropriate cell yield for the implantation (Marlovits et al., 2006; Kuroda et al., 2011). Depending on the autologous cartilage grafts, this solution significantly helps increase a large amount of chondrocytes at the cartilage damage site to facilitate the healing and repair process (Marlovits et al., 2006). Additionally, adipose tissue is known as the most suitable stem cell supplier as it can be collected easily from a liposuction procedure, which is considered to have the fewest ethical implications and increased donation (Miana and González, 2018; Park et al., 2018). Human adipose-derived stem cells (hADSCs) can be harvested in large amounts using established protocols. It was reported that collagenase-digested adipose tissue is capable of yielding up to 5 × 104 cells per milliliter of adipose tissue in a lipoaspirate, which is roughly 100-fold higher than that of bone marrow-derived MSCs. hADSCs have been extensively demonstrated to be maintained in a stable undifferentiated state during in vitro expansion, and their capacity for trilineage differentiation, including adipogenesis, osteogenesis, and chondrogenesis, is established. Recent studies have shown that hADSCs obtained from lipoaspirate can be induced under specific conditions to promote cartilage regeneration (Wu et al., 2010; Wang et al., 2018).

Bovine pericardium, which is rich in collagen, containing mostly type I collagen, as well as glycoproteins and glycosaminoglycans (GAGs), has been applied in scaffold fabrication. Bovine pericardium was first introduced in cardiovascular surgeries and is now widely used in other medical disciplines. Bovine pericardial scaffold seeded with cells was earlier demonstrated in myocardial regeneration (Liu et al., 2016; Kameli et al., 2018; Pourfarhangi et al., 2018; Wang et al., 2018). There was a study on decellularized bovine pericardial tissue as a scaffold seeded with multilayered mesenchymal stem cells in a syngeneic Lewis rats myocardial infarction model (Wang et al., 2018). Bovine pericardial ECM seeded with hADSCs was shown to facilitate cell ingrowth and small vessel invasion in vivo (Wu et al., 2015). However, there have been limited reports on the application of bovine pericardium as a chondrocyte scaffold as well as in cartilage regeneration. Bovine pericardium patches such as Tutopatch have been widely used as envelopes containing rib fragments for augmentation in rhinoplasty in order to disguise the graft and create a more natural appearance and less visibility through the skin (Gupta et al., 2006; Nguyen et al., 2015). However, the regenerative ability of such bovine pericardium material in this application has not been reported yet. Hence, in this study, we aim to introduce bovine pericardium-derived material as a chondrocyte scaffold matrix. It was hypothesized that the treated bovine pericardium could provide an appropriate surface for the attachment, proliferation, and chondrogenic differentiation of human adipose tissue-derived stem cells in vitro.

## 2. Materials and methods

### 2.1. Fabrication of treated bovine pericardium (tBP)

Raw bovine pericardial (BP) sacs collected at a local slaughterhouse were stored in cold 1X phosphate buffer saline (PBS) (GIBCO, USA) and transported to the laboratory. Bovine pericardia were stripped of pericardial fat and loose connective tissue, and subjected to decellularization as in our previous publication (Tran et al., 2016). Briefly, the intact pieces of BP were immersed into a hypotonic solution (10 mM Tris-HCl, pH 8) (Sigma-Aldrich, USA) for 8 h, followed by decellularization in 0.15% sodium dodecyl sulfate (SDS) (Sigma-Aldrich, USA) for 12 h. The decellularized bovine pericardium matrix was further crosslinked in 0.1% glutaraldehyde (Merck, Germany) for 6 h at 4 °C. Treated membranes were double-washed in NH4Cl (Merck, Germany) for 24 h and 1X PBS for 24 h. 

### 2.2. Evaluation of tBP

#### 2.2.1. Mechanical strength 

Tested samples were prepared as strips of 5 × 2 cm for mechanical strength testing for response to stress relaxation, as well as fracture behavior. Fracture force was adjusted at an extension rate of 50 mm/min (Sung et al., 1999) by an EZ50 universal testing machine (Lloyd Instruments, UK) equipped with NEXYGENPlus material testing and data analysis software. The thickness, length, and width of test samples were measured using a digital caliper (Tacklife, USA).

#### 2.2.2. In vitro degradation

tBP samples were cut into 10-cm2 pieces, measured for initial dry weight, and incubated in 0.25 mg/mL collagenase solution (Sigma-Aldrich, USA) at 37 **°**C for 1, 6, 11, and 16 days. During the incubation, the collagenase solution was refreshed after every 2 days. Tested samples were collected at the indicated time points, washed with 1X PBS, and freeze-dried for calculating dry weight after degradation. Relative dry weight change (%) after enzymatic degradation was determined as follows: % dry weight change = (remaining dry weight of sample after degradation × 100%) / initial dry weight of sample. 

#### 2.2.3. Hydroxyproline assay

tBP samples with a size of approximately 1 × 1 cm2 were weighed and degraded in 0.25 mg/mL collagenase at 37 **°**C. After 6, 24, and 48 h of incubation, the enzyme activity was stopped by adding 0.1 mL of EDTA solution (Sigma-Aldrich, USA). The digestion supernatants were collected and hydrolyzed with 37% H2SO4 at 120 **°**C for 3 h. The amount of hydroxyproline in the supernatant was determined by a hydroxyproline kit (Sigma-Aldrich, USA) using a colorimetric method at 570 nm. The final hydrolyzation samples were neutralized and added with chloramine-T in an oxidation buffer. The solutions were left to stand at room temperature for 5 min and subsequently added with DMAB/perchloric acid/isopropanol solution for the next step of chromophore development by incubation at 60 °C for 90 min according to the manufacturer’s protocol. This chromophore was measured with a Biochrom EZ Read 400 Microplate Reader (Biochrom, USA). The hydroxyproline content in the digestion supernatant was calculated using a calibration curve obtained with standard amounts of hydroxyproline. All degradation experiments were performed in triplicate.

### 2.3. Chondrogenic differentiation of human adipose-derived stem cells (hADSCs)

The available hADSCs at the fourth passage were obtained from the Laboratory of Tissue Engineering and Biomedical Materials. hADSCs were trypsinized (trypsin-EDTA, Sigma-Aldrich, USA) and reseeded into a 35-mm petri dish (105 cells/dish). hADSCs were allowed to attach and proliferate until 80% confluence. For chondrogenic differentiation, confluent hADSCs were treated with chondrogenic medium (StemPro Chondrogenesis Differentiation Kit, GIBCO, USA) and incubated at 37 °C in 5% CO2 for the next 7 and 14 days. Chondrogenic medium was refreshed every 2 days.

### 2.4. In vitro cytotoxicity assay

The cytotoxicity of tBP was determined via the liquid extract and direct contact with hADSCs according to the ISO 10993-5 guideline. 

#### 2.4.1. Direct contact

tBP was tested for potential toxicity effect on hADSCs via direct contact, in which a latex glove was used as a positive control causing toxicity for the cells. Initially, hADSCs were plated in a 35-cm petri dish at 105 cells per dish and cultured for 24 h for cell attachment as confluence. All tested samples were stripped into specimens of 1 × 1 cm2 and placed on the confluent monolayer of hADSCs. After incubation at 37 °C for 24 h, tested samples were eliminated,****and the hADSCs were stained with 0.6% crystal violet (Sigma-Aldrich, USA) in 0.6% glutaraldehyde and examined microscopically for cellular morphology and response around the samples.

#### 2.4.2. Indirect test via liquid extract

The liquid extract of tBP was prepared by incubating the sheet in culture medium (6 cm2/mL) at 37 **°**C for 24 h according to ISO 10993-12. The liquid extract of tBP was harvested and used as the test group. Culture medium was used as a blank group. Liquid extract prepared from a latex glove was used as a positive group. Meanwhile, hADSCs were seeded into a 96-well plate with 4 × 103 cell per well and cultured for 24 h. All liquid extract samples, including the test group, blank group, and positive group, were added to the wells seeded with hADSCs and incubated for a further 24 h. The MTT assay was carried out to evaluate the relative percentage of cell viability in the test group and positive group versus the blank group. Relative percentage of cell viability was represented as relative growth rate (RGR), which was calculated based upon OD575 values according to the ISO 10993-5 protocol as RGR (%) = (OD test group / OD blank group) × 100%. Cytotoxicity level was determined according to RGR as shown in Table 1 (ISO 10993-5 (Li et al., 2011)).

**Table 1:** Cytotoxicity level according to relative growth rate (RGR, %) (ISO 10993-5, (Li et al., 2011)).

RGR (%)	≥100	75–99	50–74	24–49	1–24	0
Cytotoxicity	0	1	2	3	4	5

#### 2.4.3. MTT assay

The cultured medium was removed and washed once with 1X PBS. MTT solution (0.5 mg/mL; Sigma-Aldrich, USA) was added into each well and incubated for 4 h at 37 °C for formazan crystal development. Formazan crystals were dissolved in dimethyl sulfoxide (DMSO) (Sigma-Aldrich, USA) and read at 570 nm in a microplate reader.

### 2.5. Seeding, culture, and differentiation of hADSCs onto tBP

tBP samples were prepared in dishes of 0.5 mm in diameter and placed in a 96-well culture plate. The hADSC suspension (10 µL at 106 cells/mL) was seeded onto each tBP dish. The cells were incubated for 1 h to allow cell attachment on the tBP surface. The cell-seeded tBP dishes were transferred to a new 96-well plate and 200 µL of chondrogenic medium was added to each well and incubated at 37 °C in 5% CO2. The chondrogenic medium was refreshed every 2 days. After 7 and 14 days of culture, samples were collected and tested. 

### 2.6. Scanning electron microscopy (SEM) analysis

Samples were fixed overnight in 10% formalin and then dehydrated using a sequential ethanol series (70%, 80%, 90%, 96%, and 100% (v/v), 10 min in each). Samples were loaded onto aluminum studs, coated with gold, and examined under a scanning electron microscope (JSM-6510 JEOL, Japan).

### 2.7. RT-PCR analysis

The total cellular RNAs from all cultures after 7 and 14 days were extracted by TRIzol (Invitrogen; Thermo Fisher Scientific, USA) according to the manufacturer’s protocol. RNA was reverse-transcribed into cDNA, followed by PCR according to the manufacturer’s instructions (Agilent Technologies, USA). PCR primer designs for chondrogenic genes *COMP* and *Col10a1* are presented in Table 2 (Alves Da Silva et al., 2011). The housekeeping gene glyceraldehyde-3-phosphate dehydrogenase (*GAPDH*) was used as an internal control. The thermo cycle was conducted at 95 **°**C (1 min) and 40 cycles of 95 °C (30 s), 58 °C (30 s), and 68 °C (3 min/kb), followed by 68 °C (10 min). PCR products were detected by electrophoresis on a 2% gel at 100 V for 30 min, analyzed, and photographed by a bioimaging system (UVP, Germany).

**Table 2:** Primers used for reverse transcription-polymerase chain reaction (RT-PCR) analysis.

Gene	F-Primer sequences (5’‑3’)	R-Primer sequences (5’‑3’)
Col10a1	CACGCAGAATCCATCTGAGAAT	CGTTCAGCGTAAAACACTCCAT
COMP	AGGACAACTGCGTGACTGTG	GTGTCCTTTTGGTCGTCGTT
GAPDH	ACAGTCAGCCGCATCTTCTT	ACGACCAAATCCGTTGACTC

Col10a1: Type X-a1 collagen; COMP: cartilage oligometric matrix protein; GAPDH: glyceraldehyde 3-phosphate dehydrogenase.

### 2.8. Histological and immunohistochemical (IHC) analysis

Samples were fixed overnight in 10% formalin. Samples were washed with PBS three times and then stained with alcian blue staining (Sigma-Aldrich, USA) to detect sulfated GAG. For IHC analysis, the primary antibody anti-aggrecan (Thermo Scientific, USA) was placed on the sections overnight at 4 °C. The sections were washed in PBS and incubated with a secondary****biotinylated antibody (Thermo Scientific, USA) for 30 min at room temperature. Immunostaining was detected by streptavidin-horseradish peroxidase (Thermo Scientific, USA), followed by colorimetric detection using DAB (Thermo Scientific, USA).

### 2.9. Statistical analysis

The data obtained were processed in the Excel program and the least significant difference was calculated at a probability of P = 95% by means of differential analysis (analysis of variance - ANOVA) using Statgraphic 7.0 (University of Michigan, USA).

## 3. Results

### 3.1. tBP fabrication

**Figure 1:**
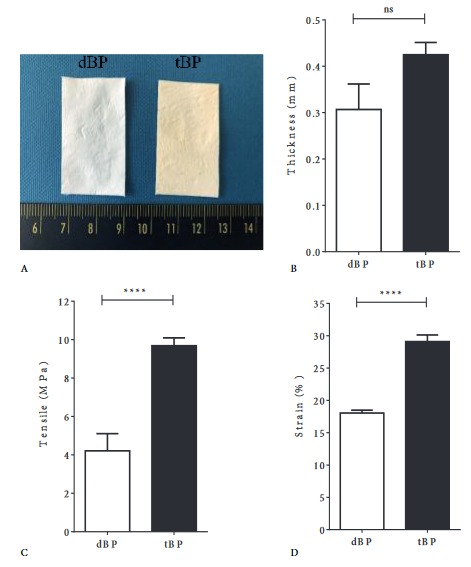
tBP fabrication and mechanical properties evaluation. A) Macroscopic observation of decellularized bovine pericardium (dBP) and treated bovine pericardium (tBP). B) Thickness (mm) measurement. C) Tensile strength (Mpa) of the samples. D) Strain (%) of the samples. ns: P > 0.05. ****: P < 0.0001.

**Figure 2:**
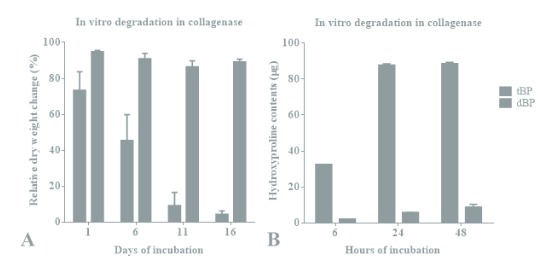
In vitro degradation of decellularized bovine pericardium (dBP) and treated bovine pericardium (tBP). A) Weight change (%) of degraded samples after 1, 6, 11, and 16 days. B) Hydroxyproline contents (μg) released from the degraded samples after 6, 24, and 48 h.

Bovine pericardium was first decellularized to achieve the acellular matrix, which was further treated by crosslinking in 0.1% glutaraldehyde for tBP fabrication. Decellularized bovine pericardium (dBP) is recognized as a thin membrane with whitish appearance. On the other hand, tBP gained a slightly yellowish color appearance due to the crosslinking procedure (Figure 1A). The thickness of tBP samples was not significantly different in comparison with dBP samples (Figure 1B). However, the tensile strength (Figure 1C) and strain property (Figure 1D) of tBP were considerably improved after crosslinking. Due to the effect of glutaraldehyde in enhancing the stability of the sheet under enzymatic conditions, the appearance and dry weight of the tBP samples remained similar with only minor changes in collagenase solution after up to 16 days of incubation (Figure 2A, gray columns). The dBP sample was found to be strongly degraded in the same incubation duration (Figure 2A, black columns). Additionally, the hydroxyproline assay determines the biodegradation of material with a more precise rate via hydroxyproline as a collagen degradation product. In the glutaraldehyde non-crosslinked group, hydroxyproline yields increased over the investigated time points for up to 24 h (Figure 2B, black columns). In the glutaraldehyde crosslink group of fabricated tBP, although there was no significant change in dry weight loss, a minor amount of hydroxyproline was found in the 24-h digestion solution (Figure 2B, gray columns), which indicated a slow degradation of tBP in enzymatic conditions and suggested that tBP may support long-term grafting in vivo and may generate within an appropriate period of time for cartilage repair. 

### 3.2. Chondrogenic differentiation

**Figure 3:**
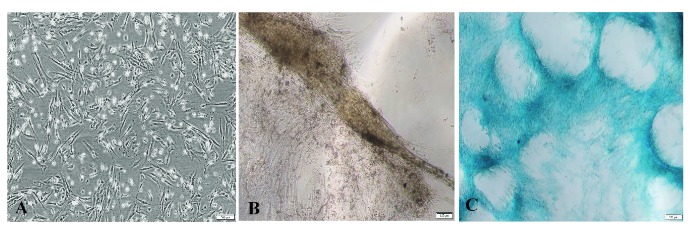
Culture and differentiation of human adipose-derived stem cells (hADSCs). A) Cultured hADSCs at 4th passage. B) Chondrocyte-like cells’ differentiation. C) Alcian blue staining for chondrogenic differentiation of hADSCs. 100× magnification. Scale bar: 100 μm.

The hADSCs were from an available cell line and were isolated, cultured, and expanded according to the protocol described previously (Tran et al., 2013). hADSCs were determined to positively express CD13, CD44, CD73, CD90, CD105, and CD166, and they differentiated into adipocytes after culturing for 21 days in adipogenic medium (Tran et al., 2013). In this study, hADSCs at the 4th passage were used. The cells adhered to the culture surface and appeared to be homogeneous in spindle-shaped morphology (Figure 3A). After chondrogenic induction for 14 days, hADSCs expressed a chondrocyte-like morphology. The cells lost their typical morphology and compacted to form large multilayered aggregates (Figure 3B). Alcian blue staining also showed the accumulation of proteoglycans in the extracellular matrix (Figure 3C).

### 3.3. In vitro cytotoxicity of tBP on hADSCs

#### 3.3.1. Direct contact test

**Figure 4:**
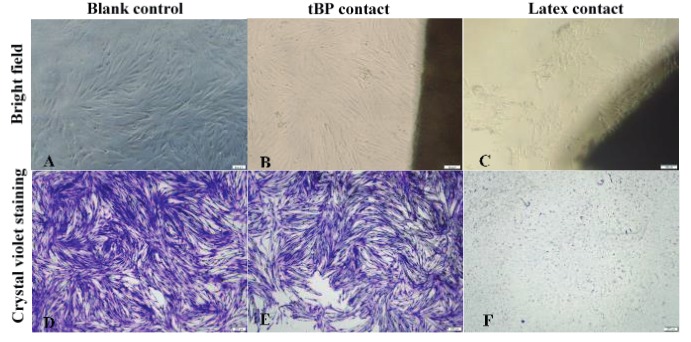
Direct contact test and crystal violet staining. A, D) hADSCs were incubated in growth medium. B, E) hADSCs were incubated in liquid extract of tBP. C, F) hADSCs were incubated in liquid extract of latex. 100× magnification. Scale bar: 100 μm.

tBP samples were examined for toxicity to hADSCs by a direct contact test, in which the materials were placed on the confluent monolayer of hADSCs. A latex membrane was used as a positive control. After 24 h of incubation, as shown in Figure 4, the neighboring hADSCs around the tBP presented a morphology similar to that cultured in growth medium (as blank control) without detachment or formation of empty space around the sample (Figures 4A and 4B). In contrast, hADSCs exposed to the latex membrane were highly necrotic with irregular shape and floating cells (Figure 4C). Crystal violet staining was also used to visualize cell morphology with high confluence in both the blank control and tested tBP sample (Figures 4D and 4E). However, it showed an empty surface with unstained cell debris, which indicated complete cell death in the positive group (Figure 4F). 

#### 3.3.2. Cytotoxicity testing on extract

**Figure 5:**
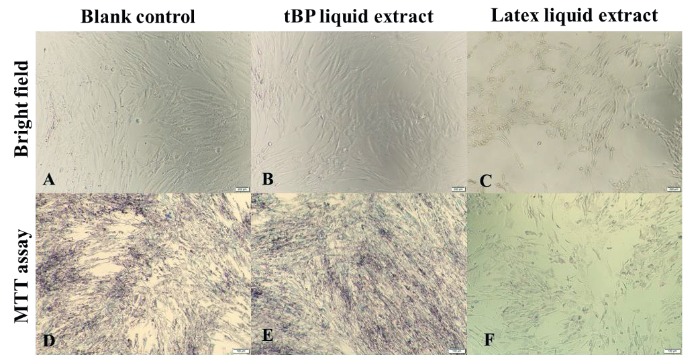
Indirect testing via liquid extract and MTT assay. A, B, C) hADSC morphology after 24-h incubation in growth medium as blank control. B) hADSC morphology after 24-h incubation in tBP liquid extract. C) hADSC morphology after 24-h incubation in latex liquid extract. D, E, F) Formazan crystal formation after MTT assay for the experimental groups. 100× magnification. Scale bar: 100 μm.

**Figure 6:**
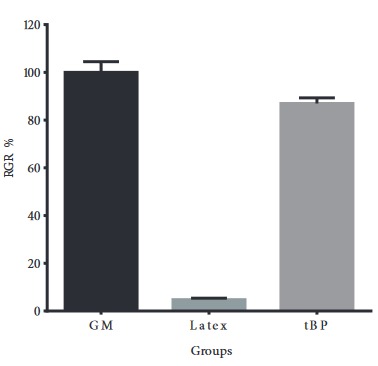
Relative growth rate (RGR, %) of tested materials. GM: Growth medium. Latex: Latex liquid extract. tBP: Treated bovine pericardium liquid extract.

There was no cell death or abnormal morphology in the groups incubated with growth medium as a blank control (Figure 5A) and tBP liquid extract (Figure 5B). However, the liquid extract of latex, as a positive control, caused serious cell detachment and death (Figure 5C). After the incubation with MTT solution, the formation of insoluble formazan crystals reflected the number of viable cells present. A dense formation of formazan crystals was detected in the blank control group and tBP liquid extract group (Figures 5D and 5E). In the positive control group, there was minor amount of formazan crystal (Figure 5F). Relative growth rate (RGR, %) of hADSCs incubated with tBP liquid extract was determined as 86.97 ± 2.37% (level 1, according to Table 1) compared to the latex liquid extract, which had a low RGR at 4.77 ± 0.60% (level 4, according to Table 1) (Figure 6). Taken together, these results demonstrated that the tBP samples showed adequate biocompatibility and could be used as an in vitro scaffold for hADSCs.

### 3.4. Cell adherence and proliferation 

**Figure 7:**
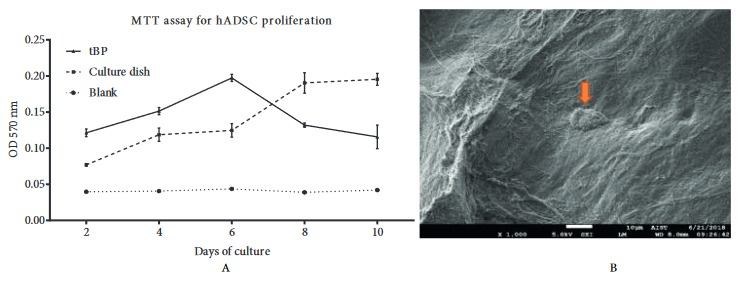
hADSCs cultured on treated bovine pericardium (tBP). A) Growth curves of hADSCs on tBP. B) Scanning electron microscope images illustrate the attachment morphology of hADSCs on tBP. Orange arrow indicates cell attachment. 1000× magnification. Scale bar: 10 μm.

Growth curves showed that the hADSCs vigorously proliferated on tBP from day 2 to 6, with the highest rate on day 6, then rapidly decreased to day 10 (Figure 7A). In addition, tBP showed the ability to support hADSC attachment after 24 h of culturing (Figure 7B). This result shows that hADSCs were capable of adhering and reached the maximum level of proliferation on tBP on day 6.

### 3.5. Extracellular matrix production

**Figure 8:**
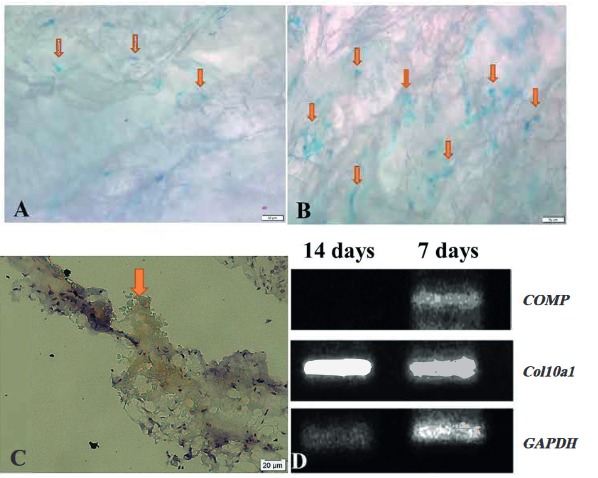
Chondrogenic differentiation of hADSC-treated bovine pericardium (tBP). A) Alcian blue staining for cell-seeded tBP at 7 days. B) Alcian blue staining for cell-seeded tBP at 14 days. C) Immunochemical staining (for aggrecan detection) of cell-seeded tBP. D) RT-PCR results express cartilage-specific gene. Orange arrows indicate the accumulation of proteoglycans. 200× magnification. Scale bar: 50 μm.

Positive alcian blue stainings were seen in the histological sections of the cell-seeded tBP on days 7 (Figure 8A) and 14 (Figure 8B). A clear difference between the 7-day and 14-day samples was the proteoglycan density within tBP. The expression of aggrecan in the cell-seeded scaffold was also demonstrated by immunochemical staining (Figure 8C). The results of alcian blue and IHC staining suggested that seeded hADSCs were differentiated into chondrocyte-like cells, which maintained their ability to synthesize and secrete a cartilage matrix after long-term culture.

### 3.6.RT-PCR

Cartilage oligomeric matrix protein (*COMP*) is present during the earliest stages of chondrogenesis. On the contrary, type X collagen is found in the transition from articular cartilage (Xu et al., 2008) to bone and is the hallmark hypertrophic chondrocyte marker. After 7 days of culture, a complex of chondroblasts (expression of *COMP*) and hypertrophic chondrocyte (expression of *Col10a1*) existed on the tBP scaffold. However, until 14 days of culture, the only hypertrophic chondrocyte (expression of *Col10a1*) existed on tBP (Figure 8D).

## 4. Discussion

Rhinoplasty is not only considered as plastic surgery for improving aesthetic appearance, but also for the correction of physical defects (Nguyen et al., 2015). Therefore, reconstruction and regeneration issues have always been considered. In addition to the well-known and widely used grafts derived from autologous and allogeneic origins, various sources and types of materials have been developed, including xenografts and alloplasts (Herman and Strauch, 2008; Malone and Pearlman, 2015), due to their abundant availability, which is one of the optimal features for ideal graft fabrication. To prevent an adverse inflammatory response, xenografts are required to undergo a decellularization procedure to eliminate cellular antigens. The resulting extracellular matrix is the final product that contributes to scaffold or material fabrication. The ECM scaffold provides a temporary habitat due to its similar components and structure to that of the native tissue. Hence, it promotes cell development. This study has shown that ECM material seeded with chondrocytes had a positive effect in facilitating the formation of neocartilage components. Thousands of publications have addressed the use of bovine pericardium ECM for biomedical materials, mostly in cardiovascular repair. Abundant quantity, rich collagen and GAG components, and excellent mechanical properties are considered to be the benefits of this type of tissue source. Recent publications have focused on the use of bovine pericardium as either the material or scaffold for cell delivery. However, the cellular differentiation behavior on bovine pericardium scaffold, especially for chondrogenic differentiation, has not been explored to date. Therefore, in our study, we aimed to use bovine pericardium as a membrane for augmentation rhinoplasty and as a scaffold for hADSCs for cartilage tissue engineering. 

The fabricated tBP had a slightly yellowish color due to glutaraldehyde crosslinking side effects (Figure 1A). Fortunately, this color was found to be similar to that of the skin; therefore, tBP might generate a good color for graft disguise. Cartilage grafts for nasal dorsum contour seemed to be good at the time of the implant, but in thin-skinned individuals, as the skin gets thinner with age, the surface at the graft margin was reported to be apparent within 16 months after rhinoplasty. Therefore, an additional covering layer (usually a temporal fascia graft) is required, which will be placed under the skin and over the cartilage graft in order to bolster the thickness of the skin, minimize unwanted visible contour irregularities, and create natural aesthetic lines (Shiffman and Di Giuseppe, 2013; Ishii, 2014; Park et al., 2015). The temporal fascia grafts are fairly thin (from 1.60 to 1.90 mm), resulting in a successful and long-lasting dorsal contouring in rhinoplasty (Shiffman and Di Giuseppe, 2013). However, additional operations and scars of 3–5 mm at the donor sites are certain consequences. “Off-the-shelf” products are considered to satisfy the current needs for transplantation in this case. The 0.5-mm-thick Permacol sheet (a porcine dermal crosslinked collagen implant) has been frequently used in augmentation rhinoplasty due to its pliability and good invisibility when placed below the dermis. Similarly, our tBP with a thickness within 0.5 mm (Figure 1B) would have good dorsum contour potential. The mechanical properties of the tBP were determined as about 10 MPa in tensile strength and 30% for strain (approximately 1.12 MPa in elongation) (Figures 1C and 1D), which were comparable to the mechanical strength of costal cartilage grafts (5.03 MPa in tensile strength and 1.33 MPa in elongation modulus) and septal cartilage grafts (12.42 MPa in tensile strength and 1.39 MPa in elongation modulus) (Alkan et al., 2011; Aguiari et al., 2015). This mechanical profile of our fabricated tBP provides a strong possibility for integration and holding and strengthening the grafts, as well as generating a smooth dorsal line. The fabricated tBP also had slow in vitro degradation, which was found to be less than 15% of dry weight loss after 16 days in enzymatic digestion (Figure 2). Therefore, it might support an ideal nasal contour. 

To tBP, which served as scaffolding material, we added human chondrocytes differentiated from hADSCs to investigate further formation of the chondrocyte sheet. hADSCs were shown to have chondrocyte differentiation potential (Figure 3), as well as high quantity after in vitro culturing, which demonstrated their promising application in fabricating cell sheets for clinical applications. We first confirmed tBP as a cell-friendly scaffold regarding the in vitro cytotoxicity on hADSCs (Figures 4–6), and its ability to support hADSC attachment and proliferation (Figure 7). These results about the in vitro biocompatibility of tBP demonstrate its capability to repair and regenerate tissues (Yi et al., 2019), therefore contributing to the integration and maintenance of the grafted tBP in augmentation rhinoplasty. In order to strengthen the application potential of tBP in augmentation rhinoplasty, tBP was evaluated for supporting the chondrogenic differentiation of hADSCs. Alcian blue staining at 7 and 14 days showed that there was an accumulation of a major component of the ECM proteoglycan after chondrogenic differentiation (Figures 8A and 8B). However, immunohistochemistry staining for aggrecan, a specific component of cartilage ECM, presented a minor expression of aggrecan (Figure 8C). *COMP* and *Col10a1* were determined as molecular markers related to chondrocyte phenotype and cartilage formation. RT-PCR results showed the expression of *COMP* after 7 days, whereas, *Col10a1* expression was detected on the 7th day and the 14th day (Figure 8D). *COMP* is involved in collagen fibril assembly in the early stage of chondrogenesis. *Col10a1* is considered as a hypertrophic cartilage marker in a later stage. Thus, the stable expression of *Col10a1* in our study indicated that hADSCs might approach an end-stage differentiation. The decreasing relative transcription level of *COMP* from day 7 to day 14 and increasing *Col10a1* expression were also observed in human chondrocytes when cultured in a cell culture incubator (Galeano-Garces et al., 2017). Consequently, GAG products from differentiated cells did not aggregate enough to form a large amount of aggrecan. Indeed, 14 days or even up to 21 days for the inducement for chondrogenic differentiation is not a long period for the accumulation of a large amount of aggrecan (Zhao and Detamore, 2010; Legendre et al., 2017), indicating the need for a longer-term investigation for greater cumulative levels of aggrecan. 

In conclusion, in the present study, treated bovine pericardium has been fabricated and evaluated for its role as a chondrocyte scaffold. The initial results demonstrated that the treated bovine pericardium was suitable to be used as a wrapping graft in augmentation rhinoplasty due to its favorable characteristics in color, texture, mechanical strength, and limited enzymatic digestion. The treated bovine pericardium also served as a suitable scaffold for hADSC attachment and proliferation. Additionally, in a chondrogenic induced medium, hADSCs seeded on the treated bovine pericardium were potentially differentiated and expressed specific markers of chondrocytes. Although there was minor accumulation of the GAG products over a 14-day period, which might require a longer time of in vitro inducement or even further in vivo investigation, all the results provide supportive evidence for the application of treated bovine pericardium as a cell scaffold for cartilage tissue engineering as well as for regenerative rhinoplasty.

## Acknowledgment and/or disclaimers, if any

The authors are grateful for the support offered by the University of Science, Vietnam National University, Ho Chi Minh City (Vietnam). This research is funded by Vietnam National University HoChiMinh City (VNU-HCM), under grant number B2017-18-07. The authors declare that there is no conflict of interest regarding the publication of this paper.

## Informed consent

Human adipose-derived stem cells were supplied by Laboratory of Tissue Engineering and Biomedical Materials and approved by the laboratory review board.
